# Intraoperative use of cell saver devices decreases the rate of hyperlactatemia in patients undergoing cardiac surgery

**DOI:** 10.1016/j.heliyon.2023.e15999

**Published:** 2023-05-04

**Authors:** Yenong Zhou, Chen Yang, Zhenxiao Jin, Bing Zhang

**Affiliations:** Department of Cardiovascular Surgery, Xijing Hospital, Fourth Military Medical University, Xi'an, China

**Keywords:** Cardiopulmonary bypass, Hyperlactatemia, Cell saver, Prognosis

## Abstract

**Objective:**

This study was aimed to elucidate the effect of the intraoperative cell saver (CS) on hyperlactatemia of patients who underwent cardiac surgery.

**Design:**

A sub-analysis of the CS was performed, which is a historial control trial of patients undergoing cardiac surgery.

**Setting:**

This was a retrospective single-center and not blinded study.

**Participants:**

We examined the occurrence of hyperlactatemia retrospectively in patients of CS group (n = 78) who were included in prospective trial and received valvular surgery, where CS was used during the procedure. Patients subjected to valvular surgery before February 2021 were adopted in control group (n = 79).

**Interventions:**

Arterial blood was sampled (1) before cardiopulmonary bypass, (2) during bypass (3) immediately after bypass, (4) on ICU admission and (5) every 4 h up to 24 h postoperatively.

**Measurements and main results:**

A lower incidence of hyperlactatemia (32.1% vs. 57.0%; P = 0.001) was observed in patients from the CS group. Furthermore, the blood lactate concentration was higher in control group than in CS group during CPB, post CPB, on ICU admission and lasted until 20 h after the operation. Multivariable analysis revealed that intraoperative use of CS was expected to be a protective factor against hyperlactatemia in this study (OR = 0.31, 95% CI 0.15–0.63, P = 0.001).

**Conclusion:**

Intraoperative use of a CS device was associated with a lower incidence of hyperlactatemia. Whether such device use is valuable to limiting hyperlactatemia in cardiac patients after surgery requires further evaluation in larger prospective studies.

## 1 Introduction

Excessive bleeding is more prone to occur when patients undergo open heart surgery due to its complex procedure and cardiopulmonary bypass (CPB), which results in transfusion- and hypoperfusion-related injuries to crucial organ systems [1,2]. A cell saver (CS) collects and washes the shed blood during surgery to reduce unwanted substrates, bacteriological contamination and residual heparin [3]; then, the salvaged red blood cells (sRBCs) can be reinfused into patients. Currently, CS is a popular strategy of blood conservation committed to reducing the volume of allogeneic blood products used [4,5]. In addition to its blood conservation ability, there is some evidence indicating that CS can provide more benefits to patients who underwent cardiac surgery with CPB. A trial enrolled 118 Jehovah's Witness patients undergoing CPB and confirmed that using CS as much as possible rather than transfusion of allogeneic blood significantly reduced acute kidney injury, postoperative blood loss, hospital stay and while not impacting mortality [6]. Another study showed that less lung injury after cardiac surgery was found in patients using CS [7]. A report demonstrated that managing shed blood with CS results in a clinically remarkable decrease in postoperative cognition impairment after cardiac surgery [8]. A study confirmed that intraoperative and early postoperative use of CS can decrease the prevalence of postoperative atrial fibrillation [9].

Hyperlactatemia is a well-described and relatively common complication in cardiac patients after surgery and is related to postoperative death and various complications [10,11]. Clinically, elevated blood lactate concentration is identified as a marker of global hypoxia. It was previously confirmed that sRBCs had increased concentrations of 2,3-diphosphoglycerate (2,3-DPG) compared to stored RBCs, which was thought to be associated with better oxygen supply [12]. However, whether the use of CS can decrease the occurrence of hyperlactatemia has not been reported in cardiac surgery.

## 2 Methods

### 2.1 Study population

A sub-analysis of the CS was performed, which is a prospective trial of patients undergoing cardiac surgery and registered in the Chinese Clinical Trial Registry (ChiCTR2100050367). The aim of the prospective trial was the effect of CS on postoperative infection. Two hundred and four patients scheduled for cardiac surgery with the use of Cell Saver Elite (Haemonetics, USA) from July 2021 to July 2022 were included in this study and related data were collected. We analysed patients from September 2021 to February 2022 who underwent valvular surgery, whether coronary artery bypass graft (CABG) was performed at the same time. We selected only valvular surgery patients due to the completeness of all relevant parameters. This trial has been licensed by Ethics Committee of Hospital and registered in the Chinese Clinical Trial Registry (ChiCTR2200060439). We extracted data from an electronic patient data-monitoring system that included 89 patients admitted to our centre (CS group). Consecutive 83 patients who underwent valvular surgery (with or without CABG) without the use of Cell Saver Elite (Haemonetics, USA) and met the eligibility criteria of this study between September 2020 to February 2021 before the start of this study were adopted as historical controls (control group). The inclusion criteria we formulated were as follows: (1) patients with age range 18–65 years; and (2) underwent valve surgery or CABG + valve surgery. The exclusion criteria we formulated were as follows: (1) preoperative severe anaemia (Hb＜10 g/dl); (2) preoperative severe impairment of renal function (creatinine clearance <30 ml/min), severe hepatic dysfunction (bilirubin >2.0 mg/dl); severe lung dysfunction (arterial partial pressure of oxygen to fraction of inspired oxygen (PaO_2_/FiO_2_) ≤300 mm Hg); (3) preoperative coagulation disorders; (4) preoperative infectious diseases; and (5) underwent urgent invasive interventions due to bleeding, heart or lung injury. In CS group, the lost blood during operation and the remaining autologous blood from cardiopulmonary bypass (CPB) were processed by the CS and transfused back into the patients, while CS was not used in the control group.

### 2.2 Anaesthesia and CPB management

Intravenous administration of rocuronium, sufentanil, propofol and midazolam at the beginning of anaesthesia induction, while sufentanil, pipecuronium, and midazolam were administrated to maintain anaesthesia during entire operation procedure.

All patients in this trial underwent CPB based on the Guidelines for CPB in 2019 European Adult Cardiac Surgery [13]. CPB was performed by a standard protocol with S5 heart–lung machine. CPB was prefilled with priming fluid containing sodium acetate Ringer's solution and 6% hydroxyethyl starch 130/0.4 and electrolyte injection (Fresenius AG, Bad Homburg, Germany). After splitting the sternum, CPB was established through the ascending aorta and superior and inferior vena cava. Cardioplegia was perfused through the aortic root or coronary opening.

CPB was initiated after systemic heparinization and activated clotting time (ACT) was greater than 480 s.

The perfusion flow rate was maintained at 2.4 L/(m^2^/min) and the mixed venous blood oxygen saturation was maintained above 75% under mild hypothermia conditions. During the operation, the perfusion pressure was monitored through the radial artery or femoral artery, and the mean arterial pressure was maintained at 50–80 mmHg. When nasopharyngeal temperature was 32–34 °C, cross-clamped the ascending aorta, followed by cold blood cardioplegia perfusion through the aortic root or coronary opening. When the patient's temperature had reached 36 °C, they were gradually weaned off of CPB and protamine was used at a 1:1 ratio to neutralize heparin. All cardiac patients in this trial were operated by the same surgical team.

### 2.3 Autologous blood transfusion

CS was strictly used in accordance with standard procedures. In this experiment, a Cell Saver Elite (Haemonetic, USA) was used in the operation. Heparinized saline solution with 25.000 IU of heparin in 1 L of 0.9% saline solution at a rate of 100 mL/h was used to prevent thrombogenesis during blood collection.

In the CS group, during the period of nonheparinization, shed blood from the mediastina and wound was heparinized and then drawn into the reservoir by negative pressure (<150 mmHg). After CPB, remanent blood in CPB circuit was also recycled into the reservoir. After a series of procedures including filtrate, centrifuge, wash and concentrate, the salvaged blood became sRBCs [5]. The sRBCs infused back to the patient immediately after the procedure. In the control group, during non-heparinization, shed blood from the wound and mediastina were recycled into the suction apparatus and were abandoned. During systemic heparinization, patients’ bleeding was recycled back into the CPB circuit and infused directly to the patient after CPB.

### 2.4 Data collection

Data at each point needed in our trial was collected from specific medical work station. Serial arterial blood samples were tested for lactate at the following time points: during CPB (select the max level), post CPB, ICU admission and every 4 h up to 24 h. The postoperative vasoactive-inotropic score (VIS) was calculated on the basis of all administered inotropes and vasoconstrictors, reflecting pharmacological support of the cardiovascular system (VIS = dopamine dose [mg/kg/min] + dobutamine [mg/kg/min]+100* epinephrine dose [mg/kg/min] +50* levosimendan dose [mg/kg/min]+10* milirnone dose [mg/kg/min] + 10000* vasopressin dose [U/kg/min] + 100*norepinephrine dose [mg/kg/min]) [14].

### 2.5 Outcome endpoint

We took the incidence of hyperlactatemia (lactate concentration≥4 mmol/L) from the beginning of operation until 24 h after operation as the primary endpoint. The second endpoint were the incidence of several related postoperative complications and mortality. Assessed morbidity endpoints included renal injury [an increase in the serum creatinine concentration by ≥ 26.5 μmol/L (within 48 h) or serum creatinine 1.5 times the baseline (within 7 days)] [15], acute lung injury [arterial partial pressure of oxygen to fraction of inspired oxygen (PaO_2_/FiO_2_) ≤300 mm Hg] [16], acute liver injury [total bilirubin≥ 10*upper limit of normal or daily increasing data≥17.1 μmol/L] [17] and permanent stroke.

### 2.6 Statistics

The incidence of perioperative hyperlactatemia in this trial subset almost reached 60%. We assumed a 30% reduction in hyperlactatemia incidence as a significant index, a sample size of 63 per group was required at 90% force and a level of 0.05. Finally, a sample size of 79 patients in each group was determined in consideration of a dropout rate of 20%. The Kolmogorov‒Smirnov test was processed for normal distribution. Categorical variables were described with frequencies (%) and analysed by Fisher's exact test or the ^χ2^ test. Continuous variables with normal distribution were exhibited with means [±standard deviation (SD)] and analysed by an unpaired *t*-test. Continuous variables with nonnormally distribution were described with median [interquartile range (IQR)] and compared with the Mann‒Whitney *U* test. We employed univariate logistic regression analysis to screen possible risk factors firstly. And then the factors with P < 0.1 were further analysed by forward stepwise multivariate logistic regression to definite independent harmful and protective factors of hyperlactatemia. Data are listed as odds ratios (ORs) with 95% confidence intervals (CIs). The classification of LVEF is based on guideline recommendations [18]. Time-dependent differences were analysed with two-way analysis of variance (ANOVA). Statistical analysis was performed with SPSS 26.0 (IBM, New York, NY, USA). A *P value* < 0.05 was considered statistically significant.

## 3 Results

### 3.1 Study population

**Fig. 1:**
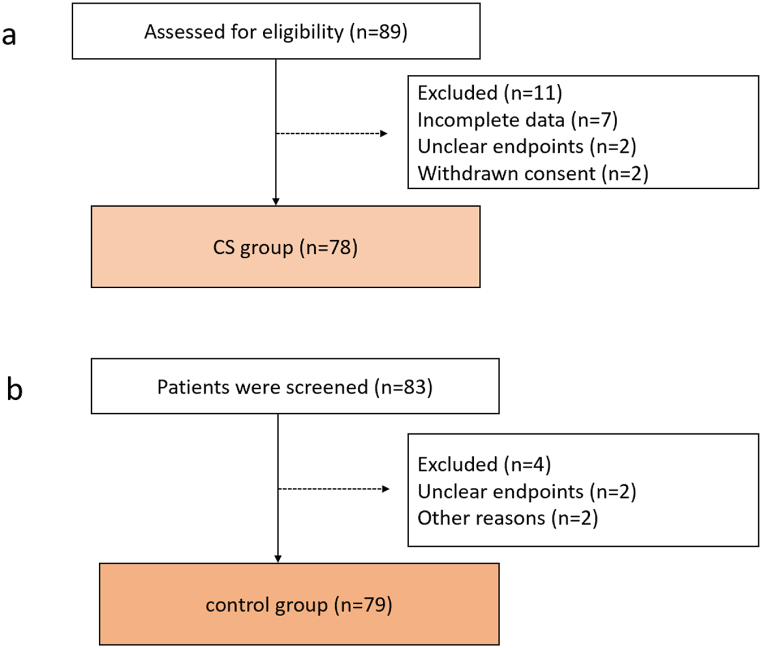
Flow chart of the study.

The enrolment flowchart of this study is shown in Fig. 1. From September 2021 to February 2022, 89 patients who underwent valvular heart disease using CS machine prospectively were enrolled in this trial. Seven patients were excluded due to incomplete data, two were excluded for lack of unclear endpoints, and two were excluded due to withdrawn consent (shown in Fig. 1a). Consecutive 83 patients without the use of CS between September 2020 to February 2021 before the start of this study were adopted in tis trial. Two patients were excluded because of unclear endpoints and two were due to other reasons (shown in Fig. 1b). The characteristics of the study subjects are listed in Table 1. No statistically significant differences were found between the groups, except for age [CS 56 (49.0–61.0) vs. 51 (40.0–60.0); *P* = 0.02].

**Table 1:** Demographic, clinical, and surgical characteristics of the CS and control groups^a^.

	CS (n = 78)	Control (n = 79)	*P*
Demographics			
Male sex	46 (59.0)	45 (57.0)	0.80
Age (y)	56 (49.0–61.0)	51 (40.0–60.0)	0.02
Weight (kg)	68 ± 10.2	65 ± 9.6	0.07
Height (cm)	168 (162.0–173.0)	167 (160.0–172.0)	0.23
Smoker	36 (46.2)	36 (45.6)	0.94
Drinker	24 (30.8)	35 (44.3)	0.08
Comorbidities			
Diabetes	22 (28.2)	20 (25.3)	0.68
Hypertension	27 (34.6)	26 (32.9)	0.82
Pulmonary disease	7 (9.0)	6 (7.6)	0.75
Previous cerebrovascular event	2 (2.6)	3 (3.8)	1.00
Stroke	1 (1.3)	3 (3.8)	0.62
History of cardiac surgery	3 (3.8)	4 (5.1)	1.00
Cardiac status			
Left ventricular function			
Moderate	23 (29.5)	26 (32.9)	0.64
Poor	2 (2.6)	3 (3.8)	1.00
NYHA			
II	16 (20.5)	16 (20.3)	0.97
III	62 (79.5)	63 (79.7)	0.97
Type of surgery			
Single valve	22 (28.2)	31 (39.2)	0.14
Multiple valves	33 (42.3)	28 (35.4)	0.38
CABG plus valve(s)	23 (29.5)	20 (25.3)	0.56
Duration of the procedure (min)	285 (250.0–362.5)	290 (255.0–335.0)	0.87
Duration of CPB (min)	136 (118.8–182.3)	131 (99.0–155.0)	0.05
Duration of aortic cross-clamping (min)	73 (60.8–92.0)	73 (59.0–90.0)	0.43
Allogeneic blood transfusion during operation			
RBC			
proportion	40 (51.3)	50 (63.3)	0.13
quantity (U)	1.5 (0–2.0)	2 (0–2.0)	0.34
Plasma			
proportion	49 (62.8)	62 (78.5)	0.03
quantity (ml)	200 (0–400.0)	350 (200.0–400.0)	0.25
Cold precipitation			
proportion	18 (23.1)	17 (21.5)	0.82
Platelet			
proportion	2 (2.6)	4 (5.1)	0.68
Preoperative HCT	43 (39.0–47.0)	45 (39.0–49.0)	0.14

^a^All normally distributed continuous variables are described as the means (SD). The median (IQR) was used for nonnormally distributed continuous variables. Categorical variables are expressed as n (%).

CABG, coronary artery bypass grafting; NYHA, New York Heart Association; HCT, haematocrit.

### 3.2 Comparison of patient outcomes

**Fig. 2:**
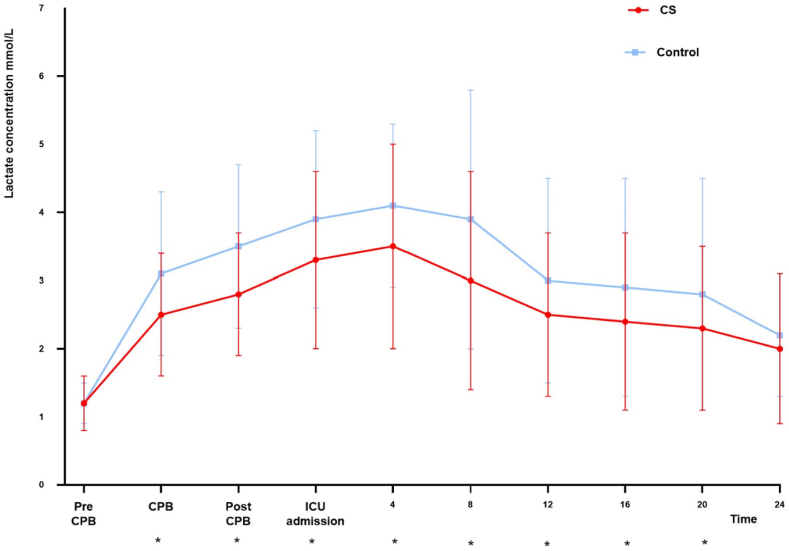
Intraoperative and postoperative lactate levels of the two groups. Mean (SD) lactate levels are shown. *Significant difference between groups (P < 0.05). CPB, cardiopulmonary bypass; ICU, intensive care unit.

The incidence of hyperlactatemia was significantly higher in the control group than in the CS group (57.0% vs. 32.1% *P* < 0.01) (shown in Table 2). The change in lactate over time between the two groups is shown in Fig. 2. The CS group had a significantly shorter ventilation time than the control group [15 h (7.7–19.2) vs. 19 h (12.3–23.9), *P* < 0.01]. Furthermore, fewer patients were exposed to allogeneic blood in the CS group, and patients in the CS group spent less on blood products (shown in Table 2).

**Table 2:** Postoperative outcomes^a^.

	CS (n = 78)	Control (n = 79)	*P*
ICU length of stay (day)	3 (2.0–4.0)	3 (2.0–4.0)	0.09
Hospital length of stay (day)	12 (10.0–14.3)	13 (10.0–16.0)	0.10
Ventilator duration (hour)	15 (7.7–19.2)	19 (12.3–23.9)	<0.01
hyperlactatemia	25 (32.1)	45 (57.0)	<0.01
Re-exploration	4 (5.1)	2 (2.5)	0.44
Acute lung injury	12 (15.4)	10 (12.7)	0.62
Renal injury	19 (24.4)	17 (21.5)	0.67
Liver injury	13 (16.7)	8 (10.1)	0.23
Stroke	2 (2.6)	1 (1.3)	0.62
30-day mortality	2 (2.6)	3 (3.8)	1.00
Allogeneic blood transfusions after surgery			
RBC			
proportion	42 (53.8)	60 (75.9)	<0.01
quantity (U)	2 (0–3.6)	2 (1.0–5.0)	0.02
Plasma			
proportion	68 (87.2)	78 (98.7)	0.01
quantity (ml)	600 (350.0–965.0)	800 (410.0–1210.0)	<0.01
Cold precipitation			
proportion	5 (6.4)	14 (17.7)	0.03
Platelet			
proportion	3 (3.8)	3 (3.8)	1
Total cost*10^5^ (¥)	1.2 (1.0–1.4)	1.3 (1.1–1.5)	0.11
Blood product cost (¥)	1740 (830–2357)	2000 (1300–3093)	0.01
VIS max	5 (4.0–7.0)	5 (4.0–8.0)	0.17
Postoperative HCT	32 (28.0–36.0)	32 (29.0–36.0)	0.90

^a^All normally distributed continuous variables are described as the means (SD). The median (IQR) was used for nonnormally distributed continuous variables. Categorical variables are expressed as n (%). VIS max, maximum vasoactive-inotropic score on the first postoperative day.

### 3.3 Predictors of hyperlactatemia

Results of the univariate analysis are exhibited in Table 3. The cut-off value for CPB duration was determined with the receiving operating curve that had the maximal sum of sensitivity and specificity. In our study, it was found to be 139.5 min. The final multivariate model determined by the stepwise forward regression procedure, 6 significant predictors CS, NYHA, hypertension, LVEF, CPB duration, and plasma in operation were included. The results are listed in Table 4. Notably, the intraoperative administration of CS was tended to be clinically favourable for patients due to its protective role against hyperlactatemia development (OR = 0.31, 95% CI 0.15–0.63, *P* < 0.001). The other independent risk factors for hyperlactatemia were as follows: LVEF <50 (OR = 4.04, 95% CI 1.54–10.58, *P* = 0.004), CPB duration ≥139.5 min (OR = 2.19, 95% CI 1.07–4.48, *P* = 0.031), and plasma in operation (OR = 1.00, 95% CI 1.00–1.01, *P* = 0.01).

**Table 3:** Univariate analysis of possible independent factors for hyperlactatemia^a^.

	Hyperlactatemia (n = 70)	Normal (n = 87)	*P*
Demographics			
Male sex	40 (57.1)	51 (58.6)	0.85
Age (y)	55 (46.8–61.0)	52 (41.0–60.0)	0.12
Weight (kg)	65.7 ± 10.6	67.2 ± 9.4	0.35
Height (cm)	167 (160.0–173.0)	168 (163.0–172.0)	0.26
Comorbidities			
Diabetes	21 (30.0)	21 (24.1)	0.41
Hypertension	29 (41.4)	24 (27.6)	0.07
History of cardiac surgery	3 (4.3)	4 (4.7)	1
Cardiac status			
Left ventricular function			
<50	20 (28.6)	10 (11.5)	0.01
NYHA			
II	9 (12.9)	23 (26.4)	0.04
III	61 (87.1)	64 (73.6)	0.04
Type of surgery			
Single valve	23 (32.9)	30 (34.5)	0.83
Multiple valves	27 (38.6)	34 (39.1)	0.95
CABG plus valve(s)	20 (28.6)	23 (26.4)	0.77
Preoperative HCT	46 (39.8–50.0)	43 (39.0–47.0)	0.03
Duration of the procedure (min)	283 (235.0–345.0)	290 (255.0–350.0)	0.62
Duration of CPB (min)	144 (115.3–184.3)	128 (102.0–156.0)	0.04
Allogeneic blood transfusion during operation			
RBC (U)	2 (0–2.0)	1.5 (0–2.0)	0.17
Plasma (ml)	370 (200.0–400.0)	270 (0–400.0)	0.01
CS (yes)	25 (35.7)	53 (60.9)	0.002
Volume of processed (ml)	580 (478.5–718.5)	669 (559.5–835.0)	0.07
Volume of salvaged (ml)	312 (213.0–411.5)	312 (220.0–445.5)	0.40
Duration of the usage (min)	320 (205.0–350.0)	280 (195.0–365.0)	0.44
VIS max	5 (4.0–8.0)	5 (4.0–7.0)	0.58

^a^All normally distributed continuous variables are described as the means (SD). The median (IQR) was used for nonnormally distributed continuous variables. Categorical variables are expressed as n (%). NYHA, New York Heart Association; HCT, haematocrit; CS, cell saver; CABG, coronary artery bypass grafting; CPB, cardiopulmonary bypass; RBC, red blood cells; VIS max, maximum vasoactive-inotropic score on the first postoperative day.

**Table 4:** Multivariable analysis determining factors associated with hyperlactatemia.

	OR	95% CI	*P* value
Cell Saver	0.31	0.15–0.63	0.001
LVEF<50	4.04	1.54–10.58	0.004
CPB duration≥139.5 min	2.19	1.07–4.48	0.031
Plasma in operation	1.00	1.00–1.01	0.01

LVEF, left ventricular ejection fraction; CPB, cardiopulmonary bypass.

## 4 Discussion

In this study, we explored the function of intraoperative CS on hyperlactatemia after cardiac surgery. We found that the use of CS was beneficial for protecting against hyperlactatemia. Moreover, analysis of dynamic test results showed that the serum lactate concentration of patients with CS decreased significantly from ICU admission to 20 h after cardiac surgery.

Cardiac surgery correlates with high mortality rates, which are caused by multitudinous complications [19]. Cardiac surgery is the most common medical scenario that requires massive transfusion for excess blood loss, which is one of the premier causes of perioperative hypoperfusion, resulting in increased morbidity and mortality induced by transfusion- and hypoperfusion-related complications [20]. Reducing allogeneic blood transfusions has always been a goal in cardiac surgery [21]. CS is recommended as category IIa to be routinely used during cardiac surgery for its contribution to a decrease of intraoperative allogeneic blood products use in the 2017 EACTS/EACTA Guidelines on patient blood management for adult cardiac surgery [22]. In our study, allogeneic blood transfusion to patients in the CS group significantly decreased, including RBCs, plasma and cold precipitation.

Hyperlactatemia occurs commonly in patients following cardiac surgery [23]. Patients who undergo cardiac surgery may experience significantly poor perfusion and hypoxia associated with CPB, vasoactive medications, and ischaemic injury. Poor perfusion and hypoxia are the leading causes of lactate accumulation [24]. Elevated serum lactate after cardiac surgery has been confirmed as a risk factor causing cardiopulmonary dysfunction, postoperative infection, renal impairment, prolonged hospital stays, and increased mortality [10,25,26]. Decreasing lactate concentrations in the early postoperative period has been confirmed to decrease hospital stay after cardiac surgery [10]. In our study, the intraoperative use of CS was proven to be a remarkable *anti*-hyperlactatemia protective factor. A study confirmed that transfusion of autologous blood, rather than allogeneic RBCs, maintained the deformability of RBCs in the circulating blood and lead to potentially better outcomes [27]. Previous studies confirmed that sRBCs has increased levels of 2,3-DPG rather than stored RBCs, indicating a better oxygen supply capacity [12,28]. Stored RBCs, but not sRBCs, had a left-shift trend in the statistical oxyhemoglobin dissociation curve. Postoperatively, 2,3-DPG levels remained a poor level below preoperative baseline for more than 3 postoperative days in patients who received stored RBCs but remained unchanged in those who received only salvaged RBCs [12]. Elevated lactate is also associated with the amount of allogeneic blood transfused [29]. In our study, allogeneic blood transfusions to patients after surgery in the CS group showed a significantly reduction compared to control group, and lower serum concentrations of lactate in the CS group might also be associated with less allogeneic blood infusion. Combined with the above, we speculated that the use of CS may reduce lactate production by improving oxygen supply to the body after cardiac surgery.

Intraoperative blood loss, CPB and cardiac insufficiency are directly related to the malignant complications of different organ systems, including the brain, liver, kidney, and gastrointestinal tract [[30], [31], [32]]. A critical reduction in perfusion or significant hypoxemia may cause hypoxic organ damage throughout the body [11]. After cardiac surgery, reduced cardiac output and excessive blood loss will cause organ function injury to varying degrees. Therefore, every organ system in the body is routinely monitored after surgery. Liver and pulmonary dysfunction are common complications after cardiac surgery because of remarkable physiological changes induced by CPB and cardiac insufficiency [33,34]. Previous research results have identified the protective role of CS in cardiac surgery on acute kidney injury, lung injury and postoperative cognitive dysfunction [[6], [7], [8]]. But in our study, we did not find that the use of CS can attenuates organ dysfunction in patients undergoing cardiac surgery.

Differences in postoperative HCT in patients were also considered because anaemia is also a direct factor affecting oxygen supply to the body [35]. We found that there was no significant difference in HCT. Furthermore, there was also no significant difference in the postoperative use of vasoactive agents, suggesting that the difference in oxygen supply between the two groups was not related to the use of vasoactive drugs, since it is well known that the use of vasoactive drugs clearly affects the blood lactate concentration of patients [36]. To some extent, we can rule out other factors that contribute to metabolic acidosis in addition to hypoxia.

Other postoperative outcomes of patients in the two groups were also observed. Post-cardiac surgery, ICU stay duration correlates closely with in-hospital mortality rates [37,38]. Some studies have shown that the use of CS was not associated with the length of ICU stay [39], they are consistent with the results of our study. It was reported that ventilator duration after surgery of patients using CS was also obviously shorter than that in patients without CS, while ventilator duration is closely related to the poor prognosis of patients [40]. Studies have shown that prolonged mechanical ventilation carries a risk of ventilator-associated infection [41], while ventilator-associated pneumonia is the most common and serious nosocomial infection that threatens patients who have undergone cardiac surgery [42]. It also increase the doses of psychotropic drugs such as sedation [43]. Prolonged mechanical ventilation (PMV) after cardiac surgery is a significant financial burden for hospitals due to the length of ICU stays and hospital stays. The vast majority of PMV patients require tracheotomy, long-term ICU care, and ultimately longer hospital stays and repeated hospitalizations. More effective ventilator management strategies are still being explored to reduce patient ventilator ventilation time [44]. Furthermore, we found that the blood products cost of the CS group were significantly lower than those of the control group. Therefore, we believe that the use of CS is potentially beneficial to postoperative outcomes.

There are also some limitations in our research. The sample size is relatively small, and whether the difference in postoperative outcomes is due to the difference in blood lactate levels between the two groups needs further analysis. According to the results of this experiment, it is known that the incidence rate of hyperlactatemia in the control group is 57%, the OR of using CS is 0.3, α = 0.05, β = 0.1, and the cases are included according to the ratio of 1:1 between the experimental group and the control group. Considering the drop-out rate of 20%, a total of 388 cases needs to be included. In the next step, we will use this sample size as a basis for verification the relationship between the use of CS and postoperative oxygen supply for patients who accepted cardiac surgery.

We will further study and clarify the mechanism of intraoperative CS use to reduce the concentration of lactate after cardiac surgery.

In summary, our study confirmed that the intraoperative use of CS was associated with reduced occurrence and degree of early postoperative hyperlactatemia.

## Statement of ethics

This trial was reviewed and approved by the Ethics Committee of Xijing Hospital, which has been registered in the Chinese Clinical Trial Registry (ChiCTR2200060439). And in accordance with national laws and institutional requirements, written informed consent was not required for this study.

## Funding

This work was supported by grants from the 10.13039/501100001809National Natural Science Foundation of China (82000227).

## Author contribution statement

Yenong Zhou: Analysed and interpreted the data; Conceived and designed the experiments; Performed the experiments; Wrote the paper.

Chen Yang: Contributed reagents, materials, analysis tools or data; Performed the experiments; Analysed and interpreted the data.

Zhenxiao Jin: Conceived and designed the experiments; Contributed reagents, materials, analysis tools or data.

Bing Zhang: Conceived and designed the experiments; Performed the experiments; Analysed and interpreted the data; Wrote the paper.

## Data availability statement

Data included in article/supp. material/referenced in article.

## Additional information

No additional information is available for this paper.

## Declaration of competing interest

The authors have no conflicts of interest to declare.
